# Fulfilled Emotional Outcome Expectancies Enable Successful Adoption and Maintenance of Physical Activity

**DOI:** 10.3389/fpsyg.2015.01990

**Published:** 2016-01-06

**Authors:** Verena Klusmann, Lisa Musculus, Gudrun Sproesser, Britta Renner

**Affiliations:** ^1^Department of Psychology, Psychological Assessment and Health Psychology, University of KonstanzKonstanz, Germany; ^2^Department of Psychology, German Sport University CologneCologne, Germany

**Keywords:** fulfillment of outcome expectancies, emotional rewards, adoption of physical activity, maintenance of physical activity, stages of behavior, longitudinal cohort study, health behavior change process

## Abstract

Although outcome expectancies are regarded as key determinants of health behavior change, studies on the role of their degree of fulfillment in long-term activity changes are lacking. This study investigated the impact of (un-)fulfilled outcome expectancies (OE) on (un-)successful attempts to increase physical activity, assuming that disengagement is the logical consequence of perceived futility. Participants (*n* = 138) of a longitudinal cohort study with three measurement waves were assigned to eight different groups according to a staging algorithm of their self-reported, 1-year-long physical activity behavior track. Stages were validated by objective changes in objective fitness, e.g., Physical Working Capacity (PWC). Social cognitive variables, self-efficacy, proximal and distal OE, and fulfillment of OE, were assessed via self-report. Discriminant analyses revealed that OE fulfillment was the predominant predictor for differentiating between successful and unsuccessful behavior change. Amongst OE, proximal OE concerning emotional rewards, in conjunction with action self-efficacy, further improved discriminatory power. OE adjustment warranting hedonic rewards appears to be a crucial mechanism as it facilitates long-term changes through interventions aimed at increasing physical activity rates. Theoretical models might benefit by including the concept of fulfilled expectations acting in terms of feedback loops between volitional and motivational processes.

## Introduction

Outcome expectancies (OE) attribute value to human action: Without expectations of resultant benefit, the likelihood of health behavior decreases. People might, for example, not question whether they could wear a helmet when riding a bicycle, but whether they decide to do so greatly depends on the belief that this is an effective tool for increasing safety. Thus, alongside self-efficacy beliefs (i.e., the self-conviction that someone is able to perform a behavior even in the face of obstacles and barriers), OE (i.e., providing the value of a behavior) are needed to enable people to form an intention to adopt a certain behavior (Bandura, 2001).

The readiness to act depends on the value of expected outcomes (e.g., Atkinson, 1957). As a core concept in enabling behavior changes, OE have long been established in common health behavior change models as, for example, perceived benefit in the Health Belief Model (Stretcher et al., 1997), behavioral beliefs in the Theory of Planned Behavior (Ajzen, 1991, 1998), or, more specifically, OE in the Health Action Process Approach (Schwarzer, 1992, 2008a). OE have the form of if-then assumptions (like “If I exercise, then I will…”). These refer to expected consequences of courses of action that differ in the degree of desire and probability (Heckhausen, 1977; Bandura, 2001). These expectations result from a contemplation process with a thorough balancing of pros and cons of anticipated behavioral outcomes. Classically, OE-values refer to positive psychological effects (e.g., fun, relaxation, companionship), body image (e.g., appearance, self-image, confidence), or health benefits (Steinhardt and Dishman, 1989; Schwarzer, 2008a).

Existing research has predominantly focused on self-efficacy beliefs, relegating OE the role of antecedents (Williams, 2010). This is surprising, given that OE were shown to be the most powerful predictor of intention in different longitudinal studies (Renner and Schwarzer, 2000), translating threat appraisals and risk perceptions into action (Renner and Schupp, 2011). Following a dynamic perspective (Renner et al., 2012a), OE are important for moving people from a pre-intending to an intending, that is, a pre-active stage of behavior change (Schwarzer, 2008a). OE are likely to remain important after individuals have formed an intention throughout the whole action process (see Fuchs, 1994). Although not specifying their direct effect on behavior, post-intentional changes of OE were assumed to determine the predictive power of the construct in the volitional phase (Schwarzer, 2008b). Specifically, proximal OE, comprising affective expectations directly related to emotional states during or directly after physical activity, predict physical activity behavior to a greater degree than distal OE, which encompass long-term consequences (Gellert et al., 2012).

In terms of a dynamic feedback loop, OE gain volitional relevance according to the degree of fulfillment: If our expectations are met, it is worth investing the effort. This loop fuels the behavior change process (Rothman, 2000; Fuchs, 2013; Loehr et al., 2014). However, the absence of desired outcomes leads to a deadlock where good intentions weaken or fade and a long-term change of habits becomes unlikely.

The World Health Organization (WHO, 2010) identified the lack of sufficient physical activity as the fourth leading risk factor for global mortality. Thus, raising physical activity levels is still an essential societal goal since for example in Germany more than 75% of the population do not meet common activity recommendations (Krug et al., 2013). Interventions designed to improve this key health behavior often fail to attain long-lasting changes in exercise behavior (e.g., Schwarzer, 2008a; Evers et al., 2012; Renner et al., 2012a). It has also been shown that affective attitude is a significant predictor of physical activity behavior (Conner et al., 2015) and positive activity experiences are important boosters for motivation and stamina (Klusmann et al., 2011). Positive exercise experiences were recently introduced as an additional predictor for changes in physical exercise levels over and above well-established social-cognitive variables (Fleig et al., 2011; Parschau et al., 2013, 2014). In line with the assumption on dynamic feedback loops, a study by Loehr and colleagues (2014) showed that daily-assessed positive physical activity experiences led to higher OE in the following week while negative experiences were followed by lower OE. Basen-Engquist and colleagues (2013) demonstrated that morning positive outcome expectancies were associated with subsequent exercise minutes at the same day, whereas negative outcome expectancies were not. Moreover, spontaneous enumeration of OE resulted in more positive OE (Rhodes and Conner, 2010). Thus, particularly positive emotional OE and their fulfillment might be important for successful adoption and maintenance of physical activity.

The aims of this study were (1) to assess behavior change trajectories concerning the adoption and maintenance of physical activity within the natural dynamics of social cognition and behavior in everyday life, (2) to validate these trajectories with objective indicators, and (3) to clarify the subjective social-cognitive dynamics of behavior change, focusing on the trajectories of those who attempted to become more physically active irrespective of the amount of objective physical activity. We assumed that if physical activity does lead to the expected outcomes, this boosts the behavior change process. In contrast, if expectancies are not met, behavior disengagement is a likely consequence.

Using longitudinal self-report activity data captured over 1 year and objective fitness parameters, the present study analyzes the dynamics between OE, OE fulfillment, and physical activity. As intended behavior initiation is critical to answering the pivotal question of whether OE come true, the target group comprised people with the attempt to become more physically active. To investigate behavior change dynamics within a real-life context, we assessed physical activity within a cohort study and in everyday life without additional interventions.

We developed a three-step staging algorithm to differentiate people with characteristic profiles of physical activity trajectories, based on classical approaches to assess behavior stages (see Prochaska et al., 1992; Schwarzer, 1992, 2008a; Weinstein and Sandman, 1992; for an overview and discussion see: Sutton, 2005). Further, we used direct measures, that is, people's self-evaluations of how successful were their attempts to translate intentions into action. This format fits with our aim to assess the social-cognitive dynamics in the behavior change processes and to clarify the role of OE fulfillment, which was similarly captured as evaluation of the degree of occurrence of previous expectations. In addition, the eight newly identified activity trajectory groups based on the staging algorithm were objectively validated by measured actual changes of physical fitness via bicycle ergometry. Finally, we examined the predictive power of the *fulfillment* of OE for successful real-life adoption and maintenance of physical activity over and above the well-established concepts of OE and self-efficacy beliefs.

## Materials and methods

### Participants

Data were collected as part of the Konstanz Life Study, a longitudinal cohort study of 1321 participants launched in spring 2012 (Renner et al., 2012b; Sproesser et al., 2015). As part of the EATMOTIVE project, the Konstanz Life Study was funded by the Federal Ministry of Education and Research (BMBF Grant 0315671, granted to BR and Harald Schupp). Participants were recruited via flyers, posters, and newspaper articles. Waves 2 and 3 took place in autumn 2012 and spring 2013. All three waves, at intervals of about 6 months, captured objective health and fitness parameters as well as self-report variables focusing on physical activity and nutrition behavior. Participants received feedback on their objective health measures referring to current norms at each measurement point.

A bicycle ergometry was used to measure objective physical fitness. Thus, it was ensured that participants' blood pressure was in the normal range, i.e., systole below 150 mm HG and diastole below 100 mm HG, that participants neither suffered from a cardiovascular disease nor a history of cardiac infarction, nor a lung disease, metabolic disorder, or a mental disorder with medical advice to avoid physical exercise, nor epilepsy or multiple sclerosis, that participants did not undergo a current anti-tumor therapy, nor had a major intervention or surgery within the last 12 months, nor suffered another severe chronic or any acute disease, and that women were not pregnant. Participants had to be at least 18 years old. There were no further inclusion or exclusion criteria for the study.

Written informed consent was obtained, and the local ethical review board (University of Konstanz) approved the study protocol. The present study focuses on data captured at Waves 2 and 3, comprising 775 (58% female) and 511 (57% female) participants, respectively. All Wave 2 participants had attended Wave 1 and all Wave 3 participants had attended Wave 2. Age ranged from 19 to 87 years, mean (*SD*) age was 47.7 (17.4) for Wave 2 and 49.8 (16.9) for Wave 3. Mean (*SD*) BMI was 24.8 (3.9) and 24.8 (3.8); years of education, including years of school and years of training, were 15.8 (2.4) and 15.8 (2.4) for Waves 2 and 3, respectively.

The drop-out sample (*n* = 264) did not differ from the remaining 511 participants regarding the study variables (all *F* < 3.64, *p* > 0.05), BMI, *F*_(1, 482)_ = 1.05, *p* = 0.31, or the sociodemographic variables (gender, years of education; both *F* < 0.47, *p* > 0.05) except age. With a mean (*SD*) age of 43.7 (17.7), drop-outs were younger than the remaining sample, *F*_(1, 771)_ = 21.96, *p* < 0.001, η^2^ = 0.03. This is due to the higher number of young participants at the onset of the study period and their dropping-out leads to a more equally distributed age of participants in the final sample.

### Measures

#### Physical activity trajectories

A three-step staging algorithm was developed to assess different physical activity trajectories. In a first step at Wave 2, baseline stage (at Wave 1) was assessed retrospectively based on established criteria and validated items for stage assignment (e.g., Sutton, 2005; Lippke et al., 2009). In a second step, at Waves 2 and 3, participants assessed their behavior since the last measurement wave (Waves 1 and 2, respectively) and stated how successful eventual attempts to increase physical activity were.

Specifically, at Wave 2, participants were asked to retrospectively indicate their regular activity level before Wave 1 of the Konstanz Life Study on a five-point scale ranging from (1) *I had been active regularly for a longer period before*, (2) *I had tried to become active but did not succeed or had been active for a short period only*, (3) *I was not active but had thought about it*, (4) *I was not active and had not even thought about it*, to (5) *I was not active since it is not necessary to be active on a regular basis*. Participants who stated that they had either been active on a regular basis (1) or had tried to become active (2) were included in this study. Non-intenders (Schwarzer, 1992, 2008a) or (pre-)contemplators (Prochaska et al., 1992), or those who decided not to act (Weinstein and Sandman, 1992), that is, people without intentions or attempts to become more active were disregarded given that they did not enter the behavior change process.

Furthermore, at Waves 2 and 3 participants indicated whether they had increased physical activity since the last measurement point. Answers to the question “Since your last participation in the Konstanz Life Study, have you been more physically active than before?” were given on a four-point scale ranging from (1) *Yes, I became more physically active*, (2) *No, but I tried to become more physically active and did not succeed*, (3) *No and I have not (even) tried*, to (4) *No, because I was already active on a regular basis before*. Again we concentrated on participants who reported option 1 or 2 because they were assumed to have entered the volitional phase and presumably underwent the dynamic process of behavior change. Accordingly, characteristic activity trajectories of people who attempted to initiate or further enhance their physical activity behavior were modeled across three steps over 1 year. This procedure resulted in eight groups (see Staging Algorithm for Classifying Participants).

#### Objective fitness status

Height and weight were measured following standardized procedures by trained research staff at all three study waves. Participants wore light indoor clothing and were asked to take off their shoes. Height was measured to the nearest 0.1 cm using a wall-mounted stadiometer. Weight was measured using a digital scale (Omron Body Composition Monitor, BF511) to the nearest 0.1 kg. BMI was calculated as weight in kilograms divided by height in meters squared (kg/m^2^).

Physical fitness was assessed at all three study waves by Physical Working Capacity (PWC) using a bicycle ergometer with pulse monitoring. The PCW refers to the physical performance of a person measured in watts at a specific heart rate (here: 130, i.e., PWC 130) divided by body weight (W/kg). The higher the PWC, the better a person's physical fitness.

#### Outcome expectancies (OE) and fulfillment of OE

Eight items were used to capture OE, based on the assessment in the Berlin Risk Appraisal and Health Motivation Study (cf. Schwarzer and Renner, 2000). Four items measured proximal OE (Cronbach's α = 0.87), focusing on emotional outcomes very closely connected with the behavior, and another four measured distal OE (Cronbach's α = 0.81), focusing on long-term gains such as health and social recognition. The eight specific items used were “If I am active on a regular basis, then…” (1) *I feel more balanced*, (2) *I feel better physically*, (3) *I feel more attractive*, (4) *I feel more powerful*, (5) *it is good for my health*, (6) *my family and friends like it*, (7) *others appreciate my willpower*, (8) *I am a good example for others*. Items 1–4 are for proximal OE, items 5–8 are for distal OE. Answers were given on a four-point Likert scale ranging from 1, *not at all* to 4, *absolutely true*. Scale mean scores were calculated.

The following introduction preceded the assessment of OE fulfillment: “Please select from the following list the three most important positive consequences that you personally had expected from being physically active. Afterwards please assess whether your expectations concerning these selected consequences did fulfill.” The item stem was “I had expected that if I am physically active, then…” followed by the same items as used in the OE measure. Participants selected the three expected outcomes that were most important to them by a tick box and rated the degree of fulfillment on a seven-point scale ranging from 1, 0%, 4, 50% to 7, 100% that appeared next to the statements. The mean of these three ratings was calculated to serve as the fulfillment score.

#### Action self-efficacy

Five items were used to measure self-efficacy (Cronbach's α = 0.84). These were based on items developed by Schwarzer and Renner (2000) as well as Scholz et al. (2005). The general question “How certain are you that you could overcome the following barriers?” was followed by specific items (e.g., “I can manage to be active on a regular basis, even if I have to rethink my entire way of physical activity”). A four-point response format ranging from 1, very uncertain to 4, very certain was used.

### Data analysis

SPSS Version 21.0 statistics software was used for data analysis. Less than 1% of the values were missing, with less than 0.5% missing after scale computation. Two multivariate outliers were identified. Since results omitting those did not differ from the complete sample analysis, the results of the latter are reported below.

Discriminant function analysis was used to identify differences between groups with characteristic trajectories of physical activity. Linear combinations of discriminant coefficients for simultaneously analyzed dependent variables (DV) are estimated as discriminant functions maximizing group differentiation. The chi-square test based on Wilk's Lambda indicates whether a function significantly discriminates between groups. Also, classification percentage can be used to assess the quality of analysis. Press's Q-value (Chan, 2005) indicates whether the discriminant functions assign cases to groups better than chance. The influence of each DV on the differentiation of groups can in turn be assessed by the standardized discriminant coefficients that are interpreted similarly to standardized regression coefficients (β). Thus, the importance of DV can be estimated for each single discriminant function. The mean influence ($\bar{\beta }$) of one DV over all functions is calculated by weighing the DV's standardized discriminant coefficient in each function with the function's eigen-value and dividing it by the number of functions considered (Tabachnick and Fidell, 2001).

A sequential technique (Tabachnick and Fidell, 2001) to test alternative models of varying complexity was used. Given our assumption that fulfillment of OE was the most influential variable for the discrimination between groups with successful vs. unsuccessful activity development, OE fulfillment was included first. Consecutively, proximal OE, action self-efficacy, and distal OE were included—first, one by one, then in different combinations—to test (via Chi-square comparison of Press's Q-values) whether they further improved group differentiation.

## Results

### Distinct physical activity trajectories over 1 year

#### Staging algorithm for classifying participants

By combining the answers to the two Wave 2 questions asking after participants' physical activity level, all participants that reported attempts to become more physically active were assigned to one of four basic characteristic activity trajectory groups: *persistent actors* (had been active at Wave 1 and had successfully increased their activity level at Wave 2), *insistent actors* (had been active at Wave 1 but did not succeed in further increasing their activity level at Wave 2), *progressive intenders* (had not been active at Wave 1 but succeeded in becoming more active at Wave 2), and *permanent intenders* (had not been active at Wave 1 and did not succeed in becoming more active at Wave 2).

These four groups could then be further differentiated according to their Wave 3 statement as either *successful*, having increased their activity level (again), or *unsuccessful*. This staging according to participants' reported trajectories of adoption and maintenance of physical activity finally resulted in eight distinct groups (Table 1).

**Table 1:** **Study variables by activity groups**.

**Activity groups**	***n***	**Fulfillment of OE (Waves 2–3)**		**Proximal OE (Wave 2)**		**Distal OE (Wave 2)**		**Action self-efficacy (Wave 2)**	
		***M* (*SD*)**	**Range**	***M* (*SD*)**	**Range**	***M* (*SD*)**	**Range**	***M* (*SD*)**	**Range**
Successful persistent actors (111)^*^	18	5.77 (0.76)	4.00–7.00	3.56 (0.55)	2.50–4.00	3.10 (0.68)	1.75–4.00	2.78 (0.51)	2.00–4.00
Unsuccessful persistent actors (112)	18	5.14 (1.13)	3.33–7.00	3.71 (0.46)	2.75–4.00	2.94 (0.58)	1.75–3.75	2.69 (0.42)	2.00–3.60
Successful insistent actors (121)	15	5.42 (0.86)	4.00–7.00	3.50 (0.43)	3.00–4.00	2.80 (0.71)	2.00–4.00	2.69 (0.52)	1.40–3.40
Unsuccessful insistent actors (122)	31	4.74 (1.00)	2.67–7.00	3.36 (0.66)	1.25–4.00	2.66 (0.63)	1.00–4.00	2.38 (0.54)	1.00–3.80
Successful progressive intenders (211)	14	5.45 (0.89)	4.00–7.00	3.54 (0.52)	2.50–4.00	2.91 (0.66)	2.00–4.00	2.61 (0.49)	1.80–3.40
Unsuccessful progressive intenders (212)	11	5.07 (0.89)	3.33–6.00	3.34 (0.63)	2.25–4.00	2.85 (0.58)	1.75–3.75	2.40 (0.46)	1.60–3.00
Successful permanent intenders (221)	15	5.12 (0.67)	4.00–6.67	3.45 (0.44)	3.00–4.00	2.53 (0.65)	1.50–4.00	2.39 (0.51)	1.80–3.60
Unsuccessful permanent intenders (222)	16	4.76 (0.82)	2.67–5.67	3.20 (0.56)	2.00–4.00	2.83 (0.78)	1.50–4.00	2.10 (0.63)	1.00–2.80

*OE, outcome expectancies; M, mean; SD, standard deviation*.

* *For activity groups, numbers in parentheses reflect the options chosen for activity level at Waves 1, 2, and 3. The first number in parentheses reflects the option chosen for T1 with 1, had been active regularly before and 2, had tried to become active but did not finally succeed. The second and third number in parentheses reflect the option chosen at T2 and T3 with 1, became more physically active and 2, tried to become more physically active but did not succeed*.

Of the 341 (44% of total) participants that were classified at Wave 2, *n* = 215 participated at Wave 3; of whom *n* = 138 attempted to become more physically active. Comparing drop-outs (*n* = 126) to returnees (*n* = 215) among the classified participants, there were no differences regarding the study variables (all *F* < 1.13, *p* > 0.05), BMI, *F*_(1, 203)_ = 1.56, *p* = 0.77, or sociodemographics (gender, years of education; both *F* < 0.18, *p* > 0.05) except for age, analogous to the main sample. Also, there was no selective drop-out of the different stage trajectories: 34.9% of *persistent actors*, 34.3% of *insistent actors*, 38.2% of *progressive intenders*, and 43.1% of *permanent intenders*, $\chi_{\left(3\right)}^{2}=1.71$, *p* = 0.63. With a mean (*SD*) age of 42.3 (16.8) compared to 47.6 (16.6), drop-outs were younger than the remaining sample, *F*_(1,338)_ = 8.07, *p* = 0.01, η^2^ = 0.02. Being representative of the whole sample, the 138 (60.1% female) participants of the activity trajectory groups ranged from 20 to 87 years of age, mean (*SD*) age was 47.8 (16.7), mean (*SD*) BMI was 25.4 (4.3), and years of education were 15.8 (2.3). The activity trajectory groups did not differ in baseline BMI or PWC scores, ranging from 23.78 (2.24) to 26.71 (5.00) and 1.93 (0.38) to 1.71 (0.39), respectively. There were no differences regarding age distributions across trajectory groups.

#### Objective validation of activity trajectory groups

To validate the staging of the participants into eight groups with different activity trajectories, the change of PWC scores between Waves 2 and 3 was used as the objective criterion for the actual increase in physical fitness. Neither absolute scores nor change scores were associated with the age of the participants. Analysis of variance revealed a significant main effect for the change in physical fitness, *F*_(7, 79)_ = 2.58, *p* = 0.02, η^2^ = 0.19. Planned contrasts showed that the groups reporting a successful increase in physical activity at Wave 3 indeed had, unlike unsuccessful groups, improved in terms of objective fitness, *F*_(1, 79)_ = 5.93, *p* = 0.02, η^2^ = 0.07. Specifically, those with a *successful* increase in physical activity (see Table 1) had a mean (*SD*) increase of PWC from 1.78 (0.48) to 1.82 W/kg (0.40), while unsuccessful groups showed a decreased mean (*SD*) PWC from 1.80 (0.44) to 1.74 W/kg (0.48) from Wave 2 to Wave 3, respectively.

The most critical groups are those of permanent intenders who intended to change their activity behavior from Wave 1 onwards, but were unsuccessful until Wave 2. Whereas one subgroup finally succeeded in implementing activity at Wave 3, the other's efforts failed again. Contrasting these two groups of *successful* versus *unsuccessful permanent intenders* also revealed a significant difference in PWC change scores, *F*_(1, 79)_ = 5.94, *p* = 0.02, η^2^ = 0.07. Those who were eventually successful showed a mean (*SD*) PWC increase from 1.73 (0.37) to 1.79 W/kg (0.34), whereas those who were continually unsuccessful showed a decreased mean (*SD*) PWC from 1.71 (0.39) to 1.55 W/kg (0.46), at Waves 2 and 3, respectively. Overall, the objective assessment of physical fitness via PWC confirmed the staging of participants, since all people classified as successful increased in physical fitness measured by PWC on the group level and all people classified as unsuccessful decreased in physical fitness on the group level. For the subgroup of *unsuccessful insistent actors* only, tendencies were not in the predicted direction. This, however, might be due to the fact that this group of people was consistently active throughout the study period despite their (not implemented) attempts to further increase activity levels.

### Discriminating successful behavior changes by fulfillment of OE

Fulfillment of OE (Wave 3) showed small to medium correlations with action self-efficacy (*r* = 0.36, *p* < 0.001), proximal OE (*r* = 0.13, *p* = 0.26), and distal OE (*r* = 0.30, *p* = 0.001), all Wave 2. Action self-efficacy correlated with neither proximal OE (*r* = 0.17, *p* = 0.06) nor distal OE (*r* = 0.05, *p* = 0.60), all Wave 2.

Overall, older participants (60+ years of age) and middle aged participants (35–60 years of age) were only slightly, and non-significantly, more likely to be unsuccessful (56 vs. 57%) in their attempts to increase physical activity than younger adults (45%), $\chi_{\left(2\right)}^{2}=3.84$, *p* = 0.147. Higher age was associated with somewhat less OE, *r* = −0.15, *p* < 0.001, and correspondingly less OE fulfillment, *r* = −0.24, *p* < 0.001. Action self-efficacy did not correlate with age.

Proximal OE concerning emotional rewards represent 79% of the OE that participants selected as personally most important. Thus, four out of five OE concerned hedonic gains closely related to the physical activity behavior itself. In terms of fulfillment, the most successful expectancies were feeling more balanced, mean (*SD*) = 5.32 (1.06), and gaining a higher physical well-being, mean (*SD*) = 5.30 (1.13).

First, we ran discriminant analysis using half year trajectories data (from Wave 1 to Wave 2; *n* = 341). Fulfillment of OE clearly discriminated the four groups (centroids in parenthesis) as being either successful, that is, *persistent actors* (0.45) and *progressive intenders* (0.28) or unsuccessful, that is, *insistent actors* (–0.25) and *permanent intenders* (–0.52), λ = 0.86, $\chi_{\left(3\right)}^{2}$= 45.43, *p* < 0.001, η^2^ = 0.78 (classification rate = 41.2%; Press's Q-value = 43.88, *p* < 0.001).

Second, for trajectories throughout 1 year (*n* = 138) degree of OE fulfillment (model 1 of the sequential discriminant function analyses, see Table 2) significantly differentiated the eight activity groups, *p* = 0.02. Including proximal OE (model 2a), action self-efficacy (model 2b), or distal OE (model 2c) did not improve group discrimination. However, simultaneously including OE fulfillment with proximal OE and action self-efficacy significantly improved discrimination (model 3). This discriminant model, $\chi_{\left(1\right)}^{2}=$ 5.14, *p* < 0.05, η^2^ = 0.86, offered the best classification rate (31.9%), and with a Press's Q of 41.09, *p* < 0.001, qualified the classification as better than chance. The further addition of distal OE (model 4) did not improve classification, neither in contrast to model 1 (Table 2) nor in contrast to model 3, $\chi_{\left(1\right)}^{2}=$ 0.50 (n.s.). The three discriminant functions of the final model (model 3) were interpreted (Table 3).

**Table 2:** **Sequential discriminant functions for activity groups**.

**Model**	**Predictors**	**Wilk's Lambda**	**Classification rate %**	**Improvement of prediction over model 1**
1	Fulfillment of OE	0.85	26.1	–
2a	Fulfillment of OE, proximal OE	0.75	29.4	$\chi_{\left(1\right)}^{2}=$ 2.25, n.s.
2b	Fulfillment of OE, action self-efficacy	0.77	28.6	$\chi_{\left(1\right)}^{2}=$ 1.33, n.s.
2c	Fulfillment of OE, distal OE	0.79	27.7	$\chi_{\left(1\right)}^{2}=$ 0.05, n.s.
3	Fulfillment of OE, proximal OE, action self-efficacy	0.70	31.9	$\chi_{\left(1\right)}^{2}=$ 5.14, *p* < 0.05
4	Fulfillment of OE, proximal OE, action self-efficacy, distal OE	0.64	30.3	$\chi_{\left(1\right)}^{2}=$ 3.20, n.s.

*Improvement of prediction was assessed using Press's Q-values, tested with the Chi-square test. OE, outcome expectancies*.

**Table 3:** **Discriminant functions including fulfillment of OE, proximal OE, and action self-efficacy to differentiate activity groups**.

**Function**	**Standardized canonical discriminant coefficients (β)**			**Variance explained %**	**Canonical *R^2^***
	**Fulfillment of OE**	**Proximal OE**	**Action self-efficacy**		
1	0.60	0.24	0.56	65.0	0.20
2	™0.65	0.89	0.11	33.0	0.11
3	0.56	0.48	™0.88	2.0	0.01

*OE, outcome expectancies*.

Taken together, all three functions significantly differentiated groups, λ = 0.70, $\chi_{\left(21\right)}^{2}=40.25$, *p* = 0.007, *R*^2^ = 0.20, but functions two and three, λ = 0.88, $\chi_{\left(12\right)}^{2}=14.62$, *p* = 0.26, *R*^2^ = 0.11, or function three alone, λ = 0.99, $\chi_{\left(5\right)}^{2}=0.89$, *p* = 0.97, *R*^2^ = 0.01, did not. The second function explained 33% extra variance not explained by the first function, but the third function did not substantially contribute to discrimination.

OE fulfillment (β = 0.60) was the predominant predictor of group differentiation in function 1, being the most meaningful function that explained the most variance. The groups *persistent actors* (group centroids 0.69 and 0.37 for those *successful* and those *unsuccessful* at Wave 3, respectively), *successful insistent actors* (centroid = 0.45), and *successful progressive intenders* (centroid = 0.40) were discriminated from *unsuccessful insistent actors* (centroid = −0.47) and *unsuccessful progressive intenders* (centroid = −0.26) as well as both groups of *permanent intenders* (centroids −0.10 and −0.78 for those finally *successful* and those still *unsuccessful* at Wave 3, respectively). Thus, the first function separates those most successful from those vastly unsuccessful. The latter subsample also includes those permanent intenders finally successful in increasing their physical activity level. Despite their eventual success, this group has a negative centroid, but it is the least negative. Arguably, this can be seen as a result of very recent success after being unsuccessful for the majority of the study period. Their increase in physical activity was probably more tentative than that of the four groups that had proven to be successful in the long run.

**Figure 1:**
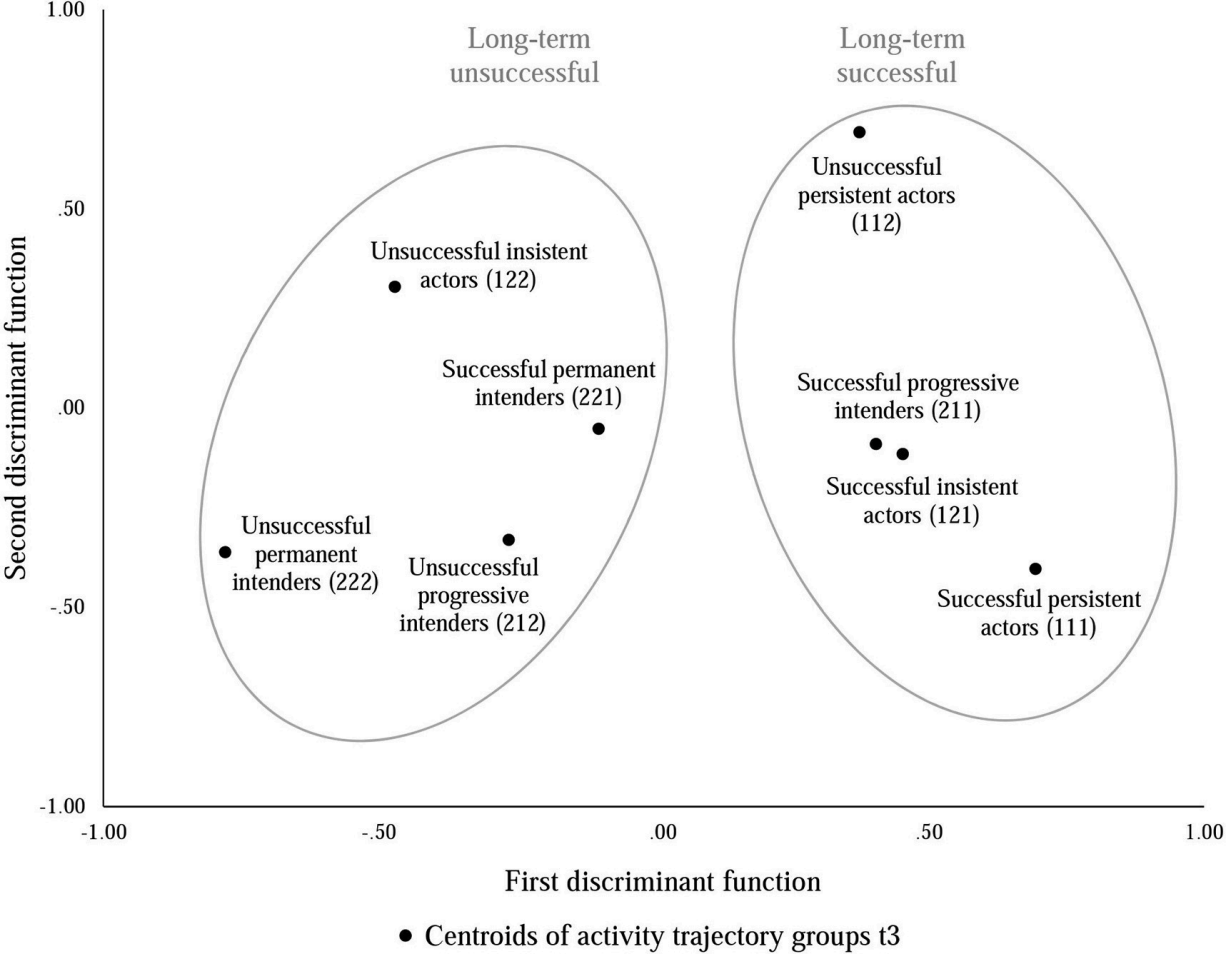
**Differentiation of activity groups according to first and second discriminant function**. The centroids of activity groups represent the mean scores of the groups on both discriminant functions that are composed of the weighed predictors according to the equations of the two discriminant functions. Ellipses surrounding centroids represent group differentiation by the first discriminant function. The long-term unsuccessful ellipse also includes successful permanent intenders. This might result from the very recent success of this group after being unsuccessful for the majority of the study period.

In function 2, the most influential factor was proximal OE (β = 0.89), discriminating *unsuccessful persistent* (centroid = 0.69) and *unsuccessful insistent actors* (centroid = 0.30) from all other groups. These groups could be described as those with the most serious relapses since both had a trajectory of successful increases of physical activity at the beginning of the study that was disrupted at Wave 3. Figure 1 shows the differentiation of the eight activity groups by the two discriminant functions. Fulfillment of OE was the most important predictor ($\bar{\beta }=0.60$) across both functions, followed by proximal OE ($\bar{\beta }=0.45$) and action self-efficacy ($\bar{\beta }=0.40$).

## Discussion

Using the longitudinal, real-life cohort sample from the Konstanz Life Study, this research indicates that the fulfillment of expectations about positive outcomes contributes significantly to successfully increasing physical activity. Groups with distinct activity trajectories were compiled using a staging algorithm based on self-reported activity levels and attempts to change behavior reported over 1 year. These groups were validated via objective fitness data. Participants who reported successful increases in physical activity indeed showed improved PWC, unlike those who did not succeed in implementing their good intentions. Activity group membership could be predicted by the reported degree of fulfillment for the three most important OE. These predominantly concerned hedonic rewards directly related to being physically active. Proximal, that is, emotional OE and self-efficacy further improved the discriminatory power to classify successful versus unsuccessful actors.

### Fulfilled OE mean positive feedback loops

Whether people report on successful increases of their activity levels seems to depend on whether their positive expectations have been met. This fits with Fuchs' (2013) and Rothman's (2000) suggestions that positive experiences are important for long-term adherence to physical activity. Obviously, if people do not get what they expected, value of the behavior is reduced and disengagement from behavior change becomes more likely. This regulation process seems by far the most logical given competing goals and limited resources, such as time investment and mental as well as physical effort. Fulfilled outcome expectancies, in contrast, reward efforts made and subsequently reinforce behavior value and thus behavior change. In a study on ceasing smoking, Baldwin and colleagues (2006) found that satisfaction with the outcomes of having quit predicted whether people maintained non-smoking status. These cycles could be visualized as positive feedback loops where hedonic rewards—since the most relevant expectations are those about proximal emotional outcomes—feed-back on good intentions that in turn reinforce activity behavior. Thus, coming from the outcomes in the volitional phase, calibration of expectancies is important for future motivational processes.

### A closer look at the dynamics of OE in health behavior models

Our study has implications for behavior change models since the fulfillment of OE was shown to be a distinct predictor that only correlates with the established social-cognitive variables OE and self-efficacy to a medium extent. Thus, future models could include expectation fulfillment to maximize power when predicting behavior change. Also, more intense data acquisition, like ecological momentary or ambulatory assessment (see Renner et al., 2015), could further help to clarify the dynamic of individual expectations and their fulfillment. Including an objective measurement of activity behavior using accelerometry, for example, would allow for an “on-line”, in-depth investigation of the interplay of OE fulfillment and actual behavior while behavior changes occur.

Our measure addressed classical domains of OE that provide the value of physical activity, being proximal affective incentives versus more distal social or health benefits (see Steinhardt and Dishman, 1989; Hallam and Petosa, 2004; Schwarzer, 2008a; Gellert et al., 2012). In analyzing the data on the three most important OE, it became evident that people had almost exclusively chosen proximal OE. This preference is advantageous given that these expectancies of positive emotional outcomes helped predict successful activity increase. Similarly, Conner and colleagues (2011) found that information on the affective outcomes of activity was more effective in increasing activity than health-related information. It seems logical that proximal OE optimally support behavior change since they are less prone to interference and are more likely to occur than distal ones. Emotional rewards directly supply hedonic needs and thus act as a driving force for behavior change maintenance. In contrast, distal outcomes like social recognition depend on the reaction of relevant others, over which the agent has little control, and whether health gains are reached is only resolved in the long run. Based on our findings, a more subtle segmenting of OE, which exceeds the dimensions of proximal and distal OE, and possibly also a detachment of expectancy (including expected likelihood of occurrence) and value could be addressed in future studies. Similarly, recent work in the physical activity domain suggested focusing on affective determinants of behavior (Rhodes et al., 2009; Jekauc et al., 2015) and systematizing different dimensions of OE (French et al., 2005; Conner et al., 2011; Gellert et al., 2012).

### Focusing on OE fulfillment in interventions

Interventions to increase physical activity have well-established the concept of action planning and coping planning (Leventhal et al., 1965; Scholz et al., 2008; Schwarzer, 2008a). In parallel to these well-established approaches to help people overcome barriers, another independent social-cognitive pathway to health behavior via satisfaction was suggested (Fleig et al., 2011). Recently, Conner and colleagues (2015) claimed studies manipulating affective attitudes and anticipated affective reactions. Extending interventions to foster realistic OE might help overcome problems of low long-term increases in physical activity (Evers et al., 2012; Renner et al., 2012a). Before becoming active, people often underestimate enjoyment because they focus on the unpleasant and aversive beginning (Ruby et al., 2011). Consequently, expectancy adjustment might help people focus on accomplishable, preferably proximal, outcomes. Also, enabling people to perceive attained positive outcomes would provide additional benefit, thereby reinforcing behavior change and ensuring behavior change maintenance. A recent proof of principle study showed that instructing exercise trainers using strategies to promote positive emotions was effective in evoking positive affective states during exercise and increasing adherence (Jekauc, 2015). Similarly, affective short-term messages produced the highest levels of physical activity in a large student sample (Morris et al., 2015). If people focus on long-term health effects or social reputation instead, they are prone to disregard emotional benefits that seem a necessary incentive for successful increases in physical activity.

### Limitations

The main limitation of our study concerns the small cell sizes that resulted from sorting people into groups with specific activity trajectories using the staging algorithm. However, the subgroups do represent the different age groups accurately and are comparable to the demographics and socio-economic status of the main sample, which was taken from a real-life cohort study. The theory-based, in-depth perspective of our data allows surveying tangible behavior change trajectories and is a major strength of our study which was undertaken in a naturalistic non-intervention setting. Still, the small number of people who are physically active or intending to become so is alarming. Like common staging procedures, we used a rating scale with verbal anchors (e.g., Sutton, 2005; Lippke et al., 2009). Although stages were validated by objective physical fitness, the subjective reasoning of behavior change is the decisive venue of social-cognitive dynamics. This is why we asked about general attempts to be “physically active regularly” without further confinement (e.g., objective recommendations for physical activity; see Haskell et al., 2007). Providing distinct goal criteria for the amount of physical activity would restrict the room for subjective goal setting. Our findings should be replicated in future studies, preferably also in health promotion programs and intervention studies that have a different respondent structure by design.

A further critique might include that the study focused on people who were already active (actors) or who had attempted to change their activity behavior (“attempters”). These numbers are comparable to similar longitudinal health screening studies (Prochaska et al., 1985; Lippke et al., 2004, 2005, 2009). As noted above, however, this also meant excluding roughly half of the participants (56%). Additional strategies are necessary to address individuals who have not thought about beginning physical activity and those who have thought about changing their behavior but not formed an intention, pre-intenders or (pre-)contemplators. To begin with the classical motivational parameters of risk information, notifying people about outcomes, and fostering self-efficacy might now, in the light of our findings, also include the promotion of outcomes with high chances of fulfillment, that is, affective rewards or emotional benefits.

Finally, we found that older participants were somewhat less successful in increasing physical activity despite actual attempts. Decreased OE and the subsequently lower OE fulfillment across age underscore that older adults are an especially critical group that needs special attention in future research and intervention design. Possibly, a maladapted profile of OE regarding physical activity in old age might contribute to the low physical activity rates in old age (Krug et al., 2013). Older people might also hold stronger negative OE and face increased physical barriers to activity than younger adults (Fuchs, 1994; Toscos et al., 2011). There is an ongoing debate on the role of negative OE (Williams et al., 2005; Schwarzer, 2008a; Conner et al., 2015). The present study focused on positive OE because spontaneous elicitation of OE resulted in more positive OE (Rhodes and Conner, 2010), and recent studies indicated that positive OE might be more important to fostering health behavior change (Aaltonen et al., 2012; Basen-Engquist et al., 2013). However, expected negative outcomes have also been found to be negatively related to health behavior changes (Williams et al., 2005; Hankonen et al., 2013; Gyurcsik et al., 2015) and might be investigated in future studies as a cause of sedentary behavior, especially in specific target groups. In line with the results on the importance of proximal, emotional OE fulfillment in our study here, we previously demonstrated that exercise in old age can indeed increase perceived emotional gains and mastery experiences derived from being physically active. This mechanism could even explain why exercise buffers aging dissatisfaction in older women (Klusmann et al., 2012). Addressing age-specificity in health behavior change dynamics seems an important issue for future studies. The development of new approaches for effective expectancy modification must cope with target-group specific dispositions.

### Conclusions

It seems that the success of sustainable behavior change depends on whether people obtain what they expect. Ascertaining whether and in how far expectations about the consequences of physical activity have been met contributes to the accurate prediction of whether people are on the road to success or doomed to failure. That the fulfillment of emotional outcome expectancies emerges as a significant predictor in addition to traditional means, namely established motivational variables, has important implications for both health behavior change modeling and intervention design.

## Funding

This research was part of the EATMOTIVE project which was funded by the German Federal Ministry of Education and Research (BMBF grant 0315671), granted to BR and Harald Schupp. The funding sources had neither an involvement in study design; in the collection, analysis, and interpretation of data; in the writing the article, nor in the decision to submit for publication.

### Conflict of interest statement

The authors declare that the research was conducted in the absence of any commercial or financial relationships that could be construed as a potential conflict of interest. The reviewer [Ines Pfeffer] declared a shared affiliation, though no other collaboration with several of the authors [VK, LM, GS, BR] to the handling Editor, who ensured that the process nevertheless met the standards of a fair and objective review.
